# Lactation performance and rumen fermentation in dairy cows fed a diet with alfalfa hay replaced by corn stover and supplemented with molasses

**DOI:** 10.5713/ajas.18.0735

**Published:** 2019-02-07

**Authors:** Zi-Hai Wei, Shu-Lin Liang, Di-Ming Wang, Hong-Yun Liu, Metha Wanapat, Jian-Xin Liu

**Affiliations:** 1 Institute of Dairy Science, College of animal Science, Zhejiang University, Hangzhou, Zhejiang 310058, China; 2 Tropical Feed Resources Research and Development Center (TROFREC), Department of Animal Science, Faculty of Agriculture, Khon Kaen University, Khon Kaen 40002, Thailand

**Keywords:** Alfalfa Hay, Corn Stover, Dairy Cows, Lactation Performance, Molasses

## Abstract

**Objective:**

The objective of current study was to investigate the lactation performance and rumen fermentation characteristics of dairy cows fed a diet with alfalfa hay replaced by corn stover but supplemented with molasses.

**Methods:**

Sixteen Holstein cows in mid-lactation were randomly assigned to 1 of 2 dietary treatments: i) alfalfa based diet (AH), and ii) corn stover based diet supplemented with molasses (CSM). The experiment was conducted according to a 2×2 crossover design with 22-d each period, consisting of 17 d for adaptation and 5 d for data and samples collection.

**Results:**

Dry matter intake and milk yield were higher for cows fed AH than CSM (p<0.01). Milk protein content and nitrogen conversion were higher (p<0.05), while milk urea nitrogen was lower (p<0.01) for cows fed AH than CSM-fed cows. Contents of milk total solids, fat and lactose were not different between two groups (p>0.10). Total rumen volatile fatty acid concentration tended to be higher (p = 0.06) for cows fed AH than CSM-fed cows. Molar proportion of acetate was lower (p = 0.04), but valerate was higher (p = 0.02) in cows fed AH than CSM-fed cows. Rumen concentration of propionate, and isobutyrate, and ratio of acetate to propionate tended to be different (p<0.10) between two groups. The feed cost per kilogram of milk was lower in CSM than AH (p<0.01). No differences were found in feed efficiency and most plasma parameters tested (p>0.10).

**Conclusion:**

In comparison with AH diet, CSM diet could be fed to dairy cows without negative effect on feed efficiency, ruminal fermentation, but economically beneficial, indicating that CSM could be an alternative choice for dairy farms instead of AH to feed mid-lactation dairy cows.

## INTRODUCTION

With the development of global dairy industry, the price of alfalfa has increased to a relatively high level, and demand for alfalfa is likely to keep on-growing in future [1]. However, alfalfa yield in its main producing regions such as United States of America tends to decline [2]. Countries such as China where alfalfa production cannot meet the dairy industry demand have to import large amount of alfalfa with high price [1–3].

Along with the corn grain production, huge amounts of corn stover (CS) are produced, representing a meaningful forage resource that can be used for ruminant livestock production. Abundant source of CS is also available for livestock in China, with over 200 million tons of CS produced annually [4,5], but these resources are not reasonably utilized and even cause severe pollution to the environment due to open burning [6,7]. Thus, it is a strategic policy for the developing countries to make full use of crop coproducts such as CS, to meet the requirement of forage and reduce the dependence on imported alfalfa hay (AH).

Non-fiber carbohydrates (NFC) including starch, sugars and pectin, are the main energy source of the diets for dairy cows [8]. Compared with alfalfa-containing diet, the diets with different forage sources but with an equivalent content of NFC and forage neutral detergent fiber (NDF) have no unfavorable effect on the ruminal fermentation and productive performance of dairy cows [9]. However, higher NFC content in alfalfa-based diet may contribute to higher feed efficiency and milk protein yield of dairy cows than in CS based diet [10,11]. In the previous study [12], we found that increasing the NFC content of CS-containing diet improved feed efficiency that reached a value similar to alfalfa-containing diet, but the feed intake was decreased, and subsequent milk yield was lower than that of AH-fed cows. Molasses is rich in sugar which could be a source of dietary NFC, and can improve diet palatability and lactation performance of dairy cows [13]. Therefore, it is hypothesized that supplementation of the CS contained diet with molasses to balance NFC content could improve lactation performance and nutrient utilization of dairy cow to a level that approaches to those fed alfalfa based diet.

## MATERIALS AND METHODS

### Animals, feeds, and design

The experiment was conducted at the Hangjiang Dairy Farm (Hangzhou, China). The Zhejiang University Institutional Animal Care and Use Committee approved all procedures involving animals (Approval No. ZJU20160375). Sixteen Holstein cows in mid-lactation (days in milk = 161±10.3) were randomly assigned to 1 of 2 dietary treatments: i) AH diet, ii) CS based diet supplemented with molasses (CSM). The experiment was conducted according to a 2×2 crossover design with 22-d each period, including 17 d for adaptation and 5 d for data and samples collection. The experimental diets were formulated according to Cornell-Penn-Miner dairy 3.0 dairy nutrition model [14] to meet the nutritional requirements of the cows. Both diets were isonitrogenous and isocaloric, and had similar NFC content (Table 1). The CS used was purchased from Yutian County, Hebei Province, which is one of the main corn grain-producing regions in China. The CS was naturally dried after corn grain collection, cut to about 5 cm long with a straw cutter, and then baled for selling. The determined chemical composition of CS and AH are listed in the footnote part of Table 1. Diets price was estimated based on the purchased price of the ingredients on farm. The feed cost per kilogram of milk was calculated by dividing the feed costs by milk yield.

All cows housed in a tie-stall barn were fed experimental total mixed ration (TMR) and had free access to drink water. The diets were offered *ad libitum* to allow at least 10% orts, and cows were fed 3 times daily at 0730, 1400, and 1930 and milked 3 times daily at 0800, 1430, and 2000, respectively.

### Sampling, measurements, and analyses

The feed offered and orts were recorded daily during d 18 to 22. The samples of TMR and orts were taken three times at each feeding time on d 20. All the samples were stored in sealed plastic bags at −20°C. The samples were dried at 65°C for 48 h, ground through a Wiley mill with 2 mm-mesh screen (Thomas-Wiley Laboratory Mill, Ramsey, MN, USA) followed by 1 mm-mesh screen (Tecator 1093, Hoganas, Sweden), and then used for chemical analysis of dry matter (DM, 105°C for 5 h), crude protein (CP, #988.05), ether extract (#920.39), crude ash (#942.05), and acid detergent fiber (ADF, #973.18) according to AOAC methods [15]. The content of NDF was analyzed according to the method of Van Soest et al [16] with α-amylase and sodium sulfite added. An ANKOM2000 fiber analyzer (Ankom Technology Corp., Macedon, NY, USA) was used to extract and filter NDF and ADF. Ingredient and chemical composition of the diets are presented in Table 1.

Milk yield and milk samples were recorded and collected daily during d 18 to 22. The samples for each day were composited proportionally (4:3:3 according to three times of milking), stored with addition of bronopol tablets (milk preservative; D & F Control System Inc., San Ramon, CA, USA), and sent to the Shanghai DHI Testing Center to analyze milk protein, fat, lactose, total solids, milk urea nitrogen (MUN) and somatic cell count (SCC) using Combi Foss FT+ instrument (Foss Electric, Hillerød, Denmark). The body weight (BW) of each cow was measured at the beginning and end of each period with a weighing scale.

On d 21 of each period, blood samples were obtained approximately 3 h after morning feeding from coccygeal artery and mammary vein, respectively, into heparinized vacuum tubes, and then centrifuged at 3,000×*g* for 15 min to obtain plasma, which was stored at −20°C for later analysis. Plasma samples were analyzed for total protein, albumin, blood urea nitrogen, glucose, nonesterified fatty acid (NEFA), and cholesterol by using Auto Analyzer 7020 instrument (Hitachi High-technologies Corporation, Tokyo, Japan) with colorimetric commercial kits (Ningbo Medical System Biotechnology Co., Ltd, Zhejiang, China).

Rumen fluid samples (approximately 100 mL) were collected using an oral stomach tube [17] after collection of blood samples. The rumen fluid pH was measured immediately using a pH meter (FE20-FiveEasy Plus; Mettler Toledo Instruments Co. Ltd., Shanghai, China). Triplicate 1-mL samples were acidified with 20 μL of orthophosphoric acid, and triplicate 1-mL original samples were frozen at −20°C for later analysis of volatile fatty acid (VFA) and ammonia nitrogen (NH_3_-N), with the methods as described by Hu et al [18].

### Statistical analysis

Data on DM intake, lactation performance, feed cost per kilogram of milk, and feed efficiency were averaged per period and analyzed using the PROC MIXED procedure of the SAS software (SAS, 2000); and the BW gain, rumen fluids variables, and blood samples parameters were also analyzed using the PROC MIXED procedure. The model included period, treatment, and treatment sequence of the animal as the fixed effects, and cow within the sequence as a random effect. The results were listed as least squares means, and were separated using the pdiff option when the fixed effects were significant. Significant difference was declared as p<0.05 and 0.05≤p≤0.10 was defined as statistical trends.

## RESULTS

### Lactation performance

Feed intake and lactation performance are presented in Table 2. The dry matter intake, milk yield and energy-corrected milk (ECM) were higher in cows fed AH than in CSM-fed cows (p<0.01). Milk protein content and nitrogen conversion (milk protein yield/CP intake) was higher (p<0.05) and MUN was lower (p<0.01) for cows fed AH compared with CSM-fed cows. No differences were found in feed efficiency (ECM/DM intake), contents of milk total solids, milk fat and lactose, SCC and BW gain between two groups (p>0.10). The feed cost per kilogram of milk was lower for cows fed CSM than for AH-fed cows (p<0.01).

### Rumen fermentation parameters

The results of rumen fermentation variables are presented in Table 3. Rumen pH tended to be lower (p = 0.06), while total VFA concentration tended to be higher (p = 0.06) for cows fed AH than CSM-fed cows. Molar proportion of acetate was lower (p = 0.04), but valerate was higher (p = 0.02) in AH than in CSM (p = 0.02). We found a tendency of difference in rumen concentration of propionate (p = 0.09), isobutyrate (p = 0.09), and ratio of acetate to propionate (p = 0.07) between two groups, with no differences in NH3-N, butyrate and isovalerate (p>0.10).

### Blood variables

No significant differences were found in the concentration of plasma total protein, albumin, glucose, NEFA and cholesterol between cows of the two groups (p>0.05), but the cows fed CSM had higher urea nitrogen in coccygeal artery (p = 0.04) and mammary vein (p = 0.02) than the AH-fed cows had (Table 4).

## DISCUSSION

Chemical composition, physical and digestion characteristics of alfalfa may cause a shorter ruminal retention time of feed particles than other forage particles [19], which may account for the higher DM intake in AH group than in CSM. Increasing energy intake can improve milk yield [20,21], thus higher DM intake in AH group supplied more energy and nutrients for milk synthesis and resulted higher milk yield in AH group than CSM. Higher supply of metabolizable protein (MP) could be expected from higher dietary content of NFC [10]. The MP supply, though not determined, might be higher in AH than in CSM in the current study, resulting in a higher milk protein content in AH (Table 2) since milk protein secretion in dairy cows is closely associated with the supply of MP [8]. The lower price of diet CSM compared with AH (Table 1) resulted in reduced feed cost per kilogram of milk in CSM, indicating higher income of each kilogram milk in CSM, which are economically beneficial for dairy farms.

Higher concentration of total VFA in AH group may be attributed to the lower NDF and higher NFC compared to the CSM. This might result in lower pH for AH group than for CSM (Table 3). Rumen NH_3_-N was not different between two groups in current study (Table 3), inconsistent with the previous finding that the cows fed CS-based diet had a higher rumen NH_3_-N than cows fed AH-based diet with no difference in pH value [11,22]. Higher pH may cause a higher rate of NH_3_-N absorption because less NH_3_-N is converted to ammonium that is absorbed more slowly than NH_3_-N [23], finally resulting in no difference of NH_3_-N between two groups (Table 3). Ammonia absorbed through the rumen wall is converted to urea in the liver and excreted in the urine and milk [24], which is reflective of higher MUN in CSM compared to AH. Higher NFC diets [25] or alfalfa based diet [26] had higher molar propionate proportion and lower acetate proportion. Though we designed to have a similar NFC content in AH and CSM in current study, the actual NFC content was a little higher in AH than CSM (Table 1), which may also contribute to the higher molar propionate proportion and lower acetate proportion in AH than CSM, and result in lower acetate: propionate in AH than in CSM (Table 3).

No differences in BW change and plasma parameters between two diets indicate no adverse effects of feeding diet CSM to lactating cows compared with AH. In combination with lower feed cost per kilogram of milk, diet CSM could be used for dairy cows to replace AH.

## IMPLICATIONS

In comparison with AH, diet CSM could be fed to dairy cows without negative effect on feed efficiency, ruminal fermentation, and blood biochemical variables, but is beneficial for dairy farms with economic merit. Our results indicate that supplementing CS diet with molasses could be an alternative choice for dairy farms instead of AH to feed mid-lactation dairy cows.

## Figures and Tables

**Table 1:** Ingredients of the experimental diets

Items	Treatment1)	

	AH	CSM
DM (%)	48.57	47.75
Dietary ingredient (g/kg of DM)		
Alfalfa hay	147	0
Oat hay	66.6	53.7
Corn stover	0	146
Corn silage	252	260
Ground corn grain	168	147
Soybean meal	130	184
Wheat bran	152	51.2
Beet pulp	71.0	92.7
Molasses	0	51.2
Premix2)	13.2	13.2
Chemical composition (g/kg of DM)		
CP	157	160
NDF	360	371
ADF	199	207
Crude ash	65	71
Ether extracts	38	34
NFC3)	379	364
NE_L_4) (MJ/kg)	7.11	7.03
Diet price (USD/kg DM)	0.343	0.319

DM, dry matter; CP, crude protein; NDF, neutral detergent fiber; ADF, acid detergent fiber; NFC, non-fiber carbohydrate; NE_L_, net energy for lactation.

1) AH, alfalfa hay based diet; CSM, corn stover based diet supplemented with molasses. Chemical compositions of the corn stover and alfalfa hay are as follows: corn stover (% of DM): 6.64% CP, 68.13% NDF, 41.45% ADF, and 9.54% ash; alfalfa hay (%of DM): 14.90% CP, 43.50% NDF, 32.76% ADF, and 8.71% ash.

2) Contained (per kilogram of DM): 540 KIU of vitamin A, 112 KIU of vitamin D, 4,500 IU of vitamin E, 1,510 mg of Fe, 1,520 mg of Cu, 7,880 mg of Zn, 1,530 mg of Mn, 50 mg of Se, 100 mg of I, 20 mg of Co, water≤10%.

3) NFC = 100–% NDF–% CP–% ether extracts–% ash (NRC [8]).

4) NE_L_ were estimated according to CPM dairy 3.0.

**Table 2:** Lactation performance in dairy cows fed experimental diets

Items	Treatment1)		SEM	p-value

	AH	CSM		
DMI (kg/d)	23.84^a^	22.55^b^	0.32	<0.01
Milk yield (kg/d)	26.61^a^	25.22^b^	1.00	<0.01
ECM2) (kg/d)	30.20^a^	28.58^b^	0.79	<0.01
Milk composition (g/kg)				
Total solid	131.8	131.3	2.00	0.35
Fat	41.05	40.88	1.41	0.74
Protein	36.18^a^	35.77^b^	0.84	0.04
Lactose	49.79	49.55	0.44	0.13
MUN (mg/dL)	17.88^b^	19.16^a^	0.62	<0.01
SCC (×10^3^) /mL	83.46	69.81	19.4	0.25
Nitrogen conversion3)	0.254^a^	0.246^b^	0.005	0.02
Feed efficiency3)	1.27	1.27	0.03	0.88
Feed cost per kg milk (USD/kg)	0.313^a^	0.286^b^	0.011	<0.01
BW gain (g/d)	493	343	130	0.43

SEM, standard error of the mean; DMI, dry matter intake; ECM, energy-corrected milk; MUN, milk urea nitrogen; SCC, somatic cell count; BW, body weight.

1) AH, alfalfa hay based diet; CSM, corn stover based diet supplemented with molasses.

2) ECM (kg) = 0.3246×milk yield (kg)+13.86×fat yield (kg)+7.04×protein yield (kg) [27].

3) Nitrogen conversion = milk protein yield/CP intake; Feed efficiency expressed as ECM (kg)/DMI (kg).

a–c Least squares means within a row with different superscripts differ (p<0.05).

**Table 3:** Ruminal fermentation variables in dairy cows fed experimental diets

Items	Treatment1)		SEM	p-value

	AH	CSM		
pH	6.16	6.31	0.07	0.06
Ammonia nitrogen (mg/dL)	17.4	18.1	1.02	0.54
Total VFA (m*M*)	107	103	2.18	0.06
Molar proportion (mmoL/100 mmoL)				
Acetate	68.80^b^	69.86^a^	0.44	0.04
Propionate	16.54	15.44	0.46	0.09
Isobutyrate	0.62	0.66	0.02	0.09
Butyrate	11.38	11.44	0.30	0.86
Isovalerate	1.37	1.41	0.10	0.72
Valerate	1.28^a^	1.18^b^	0.03	0.02
Acetate:propionate	4.23	4.57	0.13	0.07

SEM, standard error of the mean; VFA, volatile fatty acid.

1) AH, alfalfa hay based diet; CSM, corn stover based diet supplemented with molasses.

a–b Least squares means within a row with different superscripts differ (p<0.05).

**Table 4:** Plasma metabolites of dairy cows fed experimental diets

Items	Treatment1)		SEM	p-value

	AH	CSM		
Total protein (g/L)	77.65	76.78	1.36	0.13
Albumin (g/L)	26.89	26.85	0.44	0.89
Urea nitrogen (mmol/L)				
In coccygeal artery	6.38^b^	6.76^a^	0.28	0.04
In mammary vein	6.29^b^	6.82^a^	0.27	0.02
Glucose (mmol/L)				
In coccygeal artery	3.51	3.38	0.08	0.12
In mammary vein	2.53	2.42	0.09	0.26
NEFA (umol/L)	85.98	87.35	5.27	0.86
Cholesterol (mmol/L)	5.23	5.09	0.29	0.28

SEM, standard error of the mean; NEFA, nonesterified fatty acid.

1) AH, alfalfa hay based diet; CSM, corn stover based diet supplemented with molasses. The results were from mammary vein samples other than special note.
